# Can clinical signs or symptoms combined with basic hematology data be used to predict the presence of bacterial infections in febrile children under - 5 years?

**DOI:** 10.1186/s12887-018-1340-3

**Published:** 2018-11-27

**Authors:** Francois Kiemde, Massa dit Achille Bonko, Marc Christian Tahita, Palpouguini Lompo, Halidou Tinto, Petra F. Mens, Henk D. F. H. Schallig, Michael Boele van Hensbroek

**Affiliations:** 1 Institut de Recherche en Science de la Sante-Unité de Recherche Clinique de Nanoro, Nanoro, Burkina Faso; 2 Amsterdam University Medical Centers, Academic Medical Centre, Department of Medical Microbiology, Parasitology Unit, University of Amsterdam, Amsterdam, The Netherlands; 3 Global Child Health Group, Amsterdam University Medical Centers, Academic Medical Centre, University of Amsterdam, Amsterdam, The Netherlands

**Keywords:** Fever, Children, Bacteria, Malaria, Signs, Symptoms

## Abstract

**Background:**

Infectious diseases in children living in resource-limited settings are often presumptively managed on the basis of clinical signs and symptoms. Malaria is an exception. However, the interpretation of clinical signs and symptoms in relation to bacterial infections is often challenging, which may lead to an over prescription of antibiotics when a malaria infection is excluded. The present study aims to determine the association between clinical signs and symptoms and basic hematology data, with laboratory confirmed bacterial infections.

**Methods:**

A health survey was done by study nurses to collect clinical signs/symptoms in febrile (axillary temperature ≥ 37.5 °C) children under - 5 years of age. In addition, blood, stool and urine specimen were systematically collected from each child to perform bacterial culture and full blood cell counts. To determine the association between a bacterial infection with clinical signs/symptoms, and if possible supported by basic hematology data (hemoglobin and leucocyte rates), a univariate analysis was done. This was followed by a multivariate analysis only on those variables with a *p*-value *p* < 0.1 in the univariate analysis. Only a *p*-value of < 0.05 was considered as significant for multivariate analysis.

**Results:**

In total, 1099 febrile children were included. Bacteria were isolated from clinical specimens (blood-, stool- and urine- culture) of 127 (11.6%) febrile children. Multivariate logistical regression analysis revealed that a general bacterial infection (irrespective of the site of infection) was significantly associated with the following clinical signs/symptoms: diarrhea (*p* = 0.003), edema (*p* = 0.010) and convulsion (*p* = 0.021). Bacterial bloodstream infection was significantly associated with fever> 39.5 °C (*p* = 0.002), diarrhea (*p* = 0.019) and edema (*p* = 0.017). There was no association found between bacterial infections and basic haematological findings. If diarrhea and edema were absent, a good negative predictive value (100%) of a bacterial bloodstream infection was found, but the positive predictive value was low (33.3%) and the confidence interval was very large (2.5–100; 7.5–70.1).

**Conclusion:**

Our study demonstrates that clinical signs and symptoms, combined with basic hematology data only, cannot predict bacterial infections in febrile children under - 5 years of age. The development of practical and easy deployable diagnostic tools to diagnose bacterial infections remains a priority.

## Background

In resource-limited settings, infectious diseases are mainly presumptively managed on the basis of clinical signs and symptoms [1, 2]. However, the interpretation of these clinical signs and symptoms to make a diagnosis of bacterial infections is often challenging, and leads to an overuse of antibiotics in fear of overlooking bacterial infections. This practice strongly contributes to the development of drug resistance [3]. This is perhaps not the case for malaria for which rapid diagnostic tests (RDTs) are available [4]. This approach has been successful to control malaria in endemic areas [5].

However, the use of malaria RDT leaves a significant part of the febrile, non-malaria, patient population undiagnosed. Clinicians that work in areas without laboratory facilities can only manage their patients on the basis of clinical signs and symptoms, which can sometimes be supported by simple tests for hemoglobin and white blood cell count [4, 6–8]. Therefore, a proper assessment of the value of clinical signs and symptoms to predict bacterial infections could have a major practical impact. Previous studies tried to define infections according to the localization of the infection, for example chest or intestines [9–13]. However, these definitions were not focused on the infecting pathogens (bacterial, viral or parasitic). This has left a gap in fever management and explains increased numbers of antibiotic prescriptions, which is nowadays replacing the inappropriate use of anti-malarials [5]. Moreover, the interpretation of clinical signs and symptoms could vary between areas as the epidemiology of infectious diseases are different [14, 15]. For the management of febrile children, it could therefore be helpful to assess the relationship between bacterial infections and clinical signs and symptoms (supported with some simple basic hematology data).

## Methods

### Study site

The study was performed in the health district of Nanoro, located in the Center - West region of Burkina Faso at about 100 km from Ouagadougou, the capital city. The data were collected in four peripheral health facilities (Nanoro, Godo, Nazoanga and Seguedin) and at the Pediatric Department of the district referral hospital, the Centre Médical avec Antenne Chirurgicale (CMA) Saint Camille of Nanoro. The peripheral health facilities are the first medical point of contact within the community for the management of less complicated medical cases. In this setting primary health care is provided by nurses and only severe cases are transferred to the referral hospital. The referral hospital is managing the more complex cases and a pediatrician is available. Clinical signs and symptoms, and medical history, are the only information available to the attending health workers in these health facilities to make a primary diagnosis and to install disease management, except for malaria for which a rapid diagnostic test is available. Some basic laboratory data (hematology) can be made available in the referral hospital, but there is no possibility to perform for example blood cultures. Malaria is the first cause of consultation in children under - 5 years of age and occurs mainly during the rainy season which runs from July to November [16]. Vaccination against *Haemophilus influenzae* type b was introduced into the extended program of immunization (EPI) in Burkina Faso in January 2006 [16]. This program was extended with the introduction of vaccination against pneumococcal disease and rotavirus in October 2013 (Source: Ministry of Health, Burkina Faso).

### Study procedure

A cross-sectional study was conducted between January – December 2015 and April–October 2016. All children attending the pediatric service of district referral hospital or one of peripheral health facilities were routinely screened, but only children with a documented age under - 5 years and axillary temperature ≥ 37.5 °C were invited to participate in the present study. Written informed consent was obtained from accompanying parent or legal guardian. Standard Case Report Forms (CRF) were used to record clinical signs and symptoms based on clinical examination and history, together with some basic demographic information. Nurses, trained by a pediatrician and with experience in working in clinical research, performed the primary assessment of the study cases and collected the clinical signs and symptoms. The following symptoms were systematically collected by the nurses: cough, diarrhea and vomiting. The following signs were also systematically assessed during the physical examination of febrile children: edema, dehydration, jaundice, pallor of conjunctiva, bronchial crepitation’s, splenomegaly and hepatomegaly. Primary diagnosis was done according to the International Classification of Diseases (9th version) [17].

Next to the standard clinical examination, blood, stool and urine samples were systematically collected as describe previously, for cultures and full blood cell count [18]. The clinical specimens were analyzed at the microbiology laboratory of the Clinical Research Unit of Nanoro (CRUN). The microbiology laboratory of CRUN is subjected to internal quality control (according to a standard auditing protocol). Furthermore, it is also subjected to external and international quality control audits organized by the National Institute for Communicable Diseases (NICD). Bacterial bloodstream infection (BSI) or bacteremia, bacterial gastro-intestinal infection (GII) or bacterial gastroenteritis and urinary tract infection (UTI) or bacteriuria were the bacterial infections considered in this study. Children with positive bacterial culture (BSI, GII or UTI) were regrouped in general bacterial infections to assess the association between clinical signs and symptoms supported by basic hematology data with bacterial infection (all).

Patients were managed firstly according to the Burkinabe national guidelines based on WHO guidelines for the integrated management of childhood illness [19]. However, when available, the additional laboratory data were communicated to the appropriate health facilities or the district hospital as soon as these results became accessible and if needed the patient management was changed free of charge. The complementary diagnostic information and treatment provided were not used in the analysis. However, it is important to note that the outcome of diseases was not collected after the inclusion.

The study protocol was approved by the National Ethical Committee in Health Research, Burkina Faso (Deliberation N°2014–11 - 130).

### Laboratory investigations

#### Microbiological culture

Around 1–3 ml of venous blood was collected into pediatric culture bottle (BD BACTEC Peds Plus™/F, Becton Dickinson and Company, Sparks, Maryland, USA) and subsequently incubated in an automated culture system, a BACTEC 9050 instrument (Becton Dickinson), for a total of 5 days. One culture bottle was used per participant. Positive cultures were next Gram stained and further cultured on standard media like Eosin-Methylene Blue (EMB) agar, 5% Sheep Blood (SB) agar (bioMérieux, Marcy - l’Etoile, France) and chocolate gelose (CG) + isoVitalex (CG + IVX) agar, and incubated at 35–37 °C for 24 h under atmospheric conditions for EMB and at dioxide of carbon (CO_2_) for SB agar and chocolate + isoVitalex agar. Standard microbiology methods like those described in Mackie and McCartney Practical Medical Microbiology [20] and Analytical Profile Index (API) biochemical test kit (bioMerieux, France) were used to identify suspected pathogens. Contaminated blood cultures were reported as “negative blood culture”.

Fresh stool samples were screened for enterobacteriaceae - pathogens, by plating on EMB agar (only done for children under-24 months of age to check for enteropathogenic *Escherichia coli*), Hektoen agar, and sodium selenite broth, and incubated at 35–37 °C. The sodium selenite broth was sub-cultured on *Salmonella* and *Shigella* agar (SS agar) after 4 h of incubation. Suspected pathogens were identified as described above.

Collected urine was first tested with a dipstick (Standard Diagnostics, UroColor, Inc., Korea) and if positive for leucocytes and nitrite, the sample was plated on CLED (Cysteine Lactose Electrolyte Deficient) and EMB agar and incubated at 35–37 °C during 24 h. Only pure bacterial growth (i.e. only one species grown on the plate) of more than 10^5^ colonies forming units (CFU)/ml was regarded as significant bacteriuria. Bacteria count ≤10^5^ was regarded as negative and mixed growths (growth of more than one species in a sample) was regarded as contaminated and therefore disregarded. In that case, a new urine sample was not collected and considered as a missing sample. Suspected pathogens were identified with standard microbiology methods as described above.

Venous blood was collected in in ethylene-diamine tetra-acetic acid (EDTA) tubes. The full blood cell counts were assessed by using Sysmex XS1000i (Sysmex Corporation, Kobe, Japan) according to manufacturer’s instructions.

### Data analysis

Double data entry using OpenClinica software was done. Data analysis was done using R software version 3.3.1 (R Foundation for Statistical Computing, Vienna, Austria). The mean and median were used for continuous variables. For the categorical data and dichotomous variables, stratified by clinical signs and symptoms and basic laboratory data, percentage was used. In order not to miss any potential associations between clinical signs and symptoms, and basic hematology data (hemoglobin and leucocyte rates), we included all the clinical signs and symptoms, and basic hematology data in the data analysis. For the factor depending of age including weight, height and mid arm circumference, the Z - score was calculated for each child. Children with a 2 × SD for weight, height and perimeter brachial were considered as moderate malnourished and over 3 × SD like severe malnourished.

To assess whether clinical signs and symptoms can diagnose bacterial infections investigated in the present study in febrile children under - 5 years of age, the following analysis was done. Firstly, univariate logistic regression analyses were preformed to identify the subset of independent variables that were linked to general bacterial infections, as well as bacterial BSI, bacterial GII and UTI separately. In order not to miss any relevant clinical characteristics obtained at physical examination, we used all the clinical signs and symptoms reported by nurses as well as basic hematology data performed at hematology-biochemical laboratory CRUN for the univariate analysis. Only the variables with a significant level of *p* ≤ 0.1 were considered to be candidate variables for multivariate logistic regression analysis. For the determination of the association in multivariate analysis, general bacterial infections as well as bacterial BSI, bacterial GII, and UTI were adjusted for potential confounding factors (age, sex and weigh). The variables significantly associated to general bacterial infection in the univariate analyses were subsequently included in the multivariate analysis for general bacterial infections as well as bacterial BSI, bacterial GII and UTI. We calculated the association of clinical signs and symptoms, and basic hematology data with bacterial infections for the multivariate logistical regression by estimated the *p*-value (*p* ≤ 0.05). The positive and negative predictive value of the combination of the variables with significant level after the multivariate analysis were evaluated by testing these combinations in study population.

## Results

In total, 1099 febrile children under - 5 years of age were included in the study. Males represented 55.2% (607/1099) of the study population. The median age was 21 months (Interquartile [IQR]: 12–32) and 27.8% (306/1099) were children under - 12 months. The main clinical signs and symptoms reported were cough and diarrhea in 43.5% (478/1099) and 37.8% (371/1099) of the cases, respectively (Table 1). According to the z - score calculations no cases of severe malnutrition (z - score > 3SD) were found (Table 1).

**Table 1:** Clinical and basic laboratory data characteristics of study population, bacterial infected (all) and uninfected group, and bacterial infected group by infection (BSI, GII and UTI)

Characteristic	Study population	Positive and negative bacterial infection		Positive bacterial infections		
	*N* = 1099	Positive bacterial infections	Negative bacterial infections	BSI	GII	UTI
Demographic data						
Total, n (%)	1099(100.0)	127(11.6)	972(88.4)	65(5.9)	65(8.60)	11(1.5)
Male, n (%)	607(55.2)	59(46.5)	548(56.4)	34(52.3)	28(43.1)	5(45.5)
Age in months, median (IQR)	21.0 (12.0–32.0)	19.0 (12.0–25.0)	21.0 (12.0–33.0)	21.0 (13.0–30.0)	17.0 (13.0–23.0)	13.0 (7.0–21.5)
Age **≤** 12 months (%)	306(27.8)	33(26.0)	273(28.1)	16(24.6)	16(24.6)	5(45.5)
Age > 12 months (%)	793(72.2)	94(74.0)	699(71.9)	49(75.4)	49(75.4)	6(54.5)
Z-score Weight/age < 2SD in kg/month, mean (SD)	9.7(3.0)	8.9(2.4)	9.8 (3.1)	9.1(2.8)	8.9(1.9)	7.70(1.3)
Z-score Height/age < 2SD in cm/month, mean (SD)	81.0(12.3)	79.1(10.1)	82.3(12.6)	80.4(11.1)	77.4(11.7)	68.2(19.6)
Z-score MUAC in mm/age, mean (SD)	125.1(57.8)	128.9(82.2)	124.6(53.9)	132.3(109.1)	123.6(39.7)	120.0(35.8)
Admitted to referral hospital	197(17.9)	38(29.9)	159(16.4)	29(44.61)	11(16.9)	3(27.3)
Vitals						
Temperature in, **°**C, mean (SD)	38.7(0.8)	38.8(0.8)	38.7(0.8)	38.9(0.80)	38.7(0.80)	38.5(0.87)
[37.5 **°**C–38.5 **°**C], %	541(49.2)	54(42.5)	487(50.1)	26(40.0)	28(43.1)	5(45.5)
]38.5**°**-39.5 **°**C], %	394(35.9)	45(35.4)	349(35.9)	22(33.8)	26(40.0)	5(45.5)
> 39.5 **°**C (%)	164(14.9)	28(22.0)	136(14.0)	17(26.2)	11(16.9)	1(9.1)
Fever (**≥**38.5 **°**C), %	558(50.8)	73(57.5)	485(49.9)	39(60.0)	37(56.9)	6(54.5)
Respiratory rate /min, mean (SD)	38.1 (15.8)	36.7(4.9)	38.3 (16.7)	37.02 (4.4)	36.22 (5,1)	38.82 (3.6)
Laboratory data						
Hemoglobin rate, n (%)						
< 8 g/dl	300(27.3)	52(40.9)	248(25.7)	39(60.0)	18(27.7)	5(45.5)
**≥**8 g/dl- < 11 g/dl	630(57.3)	61(48.0)	569(58.9)	19(29.2)	39(60.0)	4(36.4)
≥11 g/dl	163(14.8)	14 (11.0)	149(15.4)	7(10.8)	8(12.3)	2(18.2)
No data	6(0.5)	–	–	–	–	–
White blood cells, n (%)						
< 4.10^3^ cells/mm^3^	26(2.4)	5(3.9)	21(2.2)	3(4.6)	2(3.1)	0.0
**≥**4.10^3^ - < 12.10^3^ cells/mm^3^	587(53.4)	72(56.7)	515(53.4)	32(49.2)	41(63.1)	36.4
≥12.10^3^ cells/mm^3^	478(43.5)	50(39.4)	428(44.4)	30(46.2)	22(33.8)	63.6
No data	8(0.7)	–	–	–	–	–
Signs and symptoms, n (%)						
Cough	478(43.5)	56(44.1)	422(43.4)	33(50.8)	26(40.0)	3(27.3)
Diarrhea	371(37.8)	57(44.9)	314(32.3)	29(44.6)	27(41.5)	8(72.7)
Vomiting	201(18.3)	25(19.7)	176(18.1)	11(16.9)	16(24.6)	2(18.2)
Jaundice	10(0.9)	0(0.0)	10(1.0)	0(0.0)	0(0.0)	0(0.0)
Edema	10(0.9)	5(3.9)	5(0.5)	4(6.2)	1(1.5)	0(0.0)
Pallor of conjunctiva	102(9.3)	21(16.5)	81(8.3)	18(27.7)	4(6.2)	2(18.2)
Dehydration	37(3.4)	8(6.3)	29(3.0)	6(9.2)	1(1.5)	1(9.1)
Convulsion	20(1.8)	6(4.7)	14(1.4)	4(6.2)	2(3.1)	0(0.0)

*BSI* Bloodstream infection, *GII* Gastro-intestinal infection, *UTI* Urinary tract infection, *min* minute, *mm* millimeter, *MUAC* Mid-Upper Arm Circumference

The prevalence of the bacterial infections investigated in the present study is reported in Table 1. After laboratory analyses, 1% (11/1099) of children had at least two infections at the same time (8 had BSI and GII; 2 had BSI, GII and UTI; 1 had GII and UIT). All these infections were taken into consideration whilst doing the data analysis. For bacterial BSI, *Salmonella ssp* were the most frequently isolated pathogens (78.5%; 51/65), followed by *Streptococcus pneumoniae* and *E. coli* in 6.2% (4/65) in both cases, *Staphylococcus aureus* and *Neisseria meningitides* in 3.1% (2/65) in both cases, *Haemophilus influenzae* B and *Enterobacter agglomerans* in 1.5% (1/65). For bacterial GII, enteropathogenic *E. coli* was isolated in 50.8% (33/65), *Salmonella spp* in 44.6% (29/65) and *Shigella* in 4.6% (3/65). *E. coli* was the only species isolated from UTI. Three pediatric bottle flagged positive for growth the cultures were due to contamination.

Criteria for hospital admission were not defined in the present study. The referrals and admissions were done according to the routine practice (mainly based on severity of clinical symptoms and suspected disease) In the present study, 17.9% (197/1099) of the recruited febrile children were hospitalized by health professionals. A bacterial infection was found in 3.5% (38/1099) of these children and 14.5% (159/1099) was negative for a bacterial infection. The admission rate was almost two-time higher for children with a confirmed bacterial infection 29.9% (38/127) compared to those that were negative for a bacterial infection 16.4% (159/972) (Table 1).

Table 2 shows the association of a general bacterial infection, bacterial BSI, UTI or bacterial GII, with the clinical signs and symptoms, and basic hematology data in univariate analysis. The clinical signs and symptoms significantly associated in the univariate analysis to bacterial infections were high axillary temperature (≥39.5 °C), diarrhea, dehydration, edema, convulsion, pallor conjunctiva, splenomegaly and hepatomegaly. For the basic hematology data only hemoglobin< 8 g/dl was significantly associated in the univariate analysis to bacterial infections (*p* < 0.1). Children with moderate malnutrition according to z - score calculation (weight/age and height/age) were also prone to have a general bacterial infection. Gender and age were also associated with a general bacterial infection (*p* < 0.1). Based on the information obtained from the bacterial cultures, it was found that bacterial BSI was significantly associated in the univariate analysis to diarrhea, dehydration, edema, convulsion, pallor conjunctiva, splenomegaly, hepatomegaly and hemoglobin< 8 g/dl (*p* < 0.1). Age and moderate malnutrition according to z - score calculation (weight/age and height/age) were significantly associated to UTI, after the univariate analysis. Based on stool culture, bacterial GII was associated to gender, age and moderate malnutrition according to z - score calculation (weight/age and height/age) in univariate analysis (*p* < 0.1) (Table 2).

**Table 2:** Clinical signs and symptoms and basic laboratory results associated to bacterial infection in univariate analysis

	Bacterial infected group		BSI		UTI		GII	
Clinical signs and symptoms	OR (IC 95%)	*P* value	OR (IC 95%)	P value	OR (IC 95%)	P value	OR (IC 95%)	P value
Male	1.04(1.00;1.08)	**0.034** ^a^	1.13(6.69;1.86)	0.629	1.43(0.46;4.40)	0.537	1.66(1.01;2.74)	**0.046** ^a^
Age, month, median (IQR)	1.00(1.00;1.00)	**0.050** ^a^	1.00(0.98;1.02)	0.833	0.95(0.90;1.00)	**0.069** ^a^	0.98(0.96;1.0)	**0.026** ^a^
> 12 months (%)	1.01(0.96;1.05)	0.619	1.19(0.67;2.10)	0.555	0.50(0.16;1.55)	0.227	1.19(0.67;2.10)	0.555
Z-score Weight/age < 2SD in kg/month, kg/age, mean (SD)	0.99(0.98;1.00)	**0.002** ^a^	0.93(0.85;1.02)	0.123	0.75(0.58;0.96)	**0.023** ^a^	0.90(0.82;0.98)	**0.021** ^a^
Z-score Height/age < 2SD in cm/month, cm/kg, mean (SD)	1.00(1.00;1.00)	**0.068** ^a^	1.00(0.96;1.02)	0.683	0.95(0.90;1.00)	**0.049** ^a^	0.98(0.96;1.00)	**0.072** ^a^
Z-score MUAC<2SD in mm/age mm, mean (SD)	1.00(1.00;1.00)	0.430	1.00(1.00;1.01)	0.308	1.00(0.99;1.01)	0.758	1.00(0.99;1.00)	0.821
Cough	1.00(0.97;1.04)	0.884	1.36(0.83;2.23)	0.229	0.53(0.16;1.80)	0.308	0.86(0.52;1.43)	0.563
Diarrhea	1.06(1.02;1.10)	**0.004** ^a^	1.61(0.98;2.66)	**0.061** ^a^	4.53(1.33;15.42)	**0.016** ^a^	1.41(0.85;2.34)	0.178
Dehydration	1.11(1.00;1.23)	**0.051** ^a^	3.08(1.25;7.61)	**0.015** ^a^	2.56(0.31;16.38)	0.422	0.52(0.10;2.71)	0.434
Vomiting	1.01(0.96;1.06)	0.666	0.91(0.47;0.75)	0.773	1.00(0.24;4.08)	0.993	1.48(0.83;2.65)	0.183
Edema	1.47(1.21;1.80)	**< 0.001** ^a^	9.48(2.67;33.67)	**0.001** ^a^	0.70(0.02;30.91)	0.854	1.55(0.23;10.33)	0.653
Jaundice	0.89(0.72;1.09)	**0.251**	0.35(0.02;7.30)	0.497	0.70(0.02;30.91)	0.854	0.35(0.02;7.30)	0.497
Convulsion	1.21(1.05;1.39)	**0.009** ^a^	3.74(1.23;11.40)	**0.020** ^a^	0.57(0.02;18.13)	0.752	1.65(0.40;6.77)	0.489
Pallor of conjunctiva	1.10(1.03;1.18)	**0.003** ^a^	4.20(2.34;7.52)	**< 0.001** ^a^	1,94(0.45;8.47)	0.376	0.65(0.24;1.73)	0.387
Axillary temperature [5, 38, 39]	1.01(0.97;1.05)	0.496	1.16(0.65;2.05)	0.496	1.35(0.42;4.34)	0.505	1.28(0.75;2.20)	0.900
Axillary temperature [5, 39, 42]	1.07(1.02;1.14)	**0.013** ^a^	2.23(1.19;4.18)	0.496	0.72(0.12;4.36)	0.505	1.30(0.64;2.63)	0.900
Fever (**≥**38.5 **°**C),	0.99(0.93;1.01)	0.108	0.68(0.41;1.12)	0.131	0.87(0.28;2.69)	0.812	0.77(0.47;1.28)	0.313
Bronchial congestion	0.97(0.85;1.10)	0.602	1.15(0.37;3.62)	0.807	1.96(0.28;13.72)	0.499	0.78(0.21;2.92)	0.712
Splenomegaly	1.18(1.07;1.29)	**< 0.001** ^a^	6.80(3.40;13.57)	**0.000** ^a^	0.39(0.02;8.71)	0.556	0.718(0.19;2.66)	0.620
Hepatomegaly	1.10(1.01;1.19)	**0.030** ^a^	3.50(1.69;7.23)	**0.001** ^a^	0.35(0.02;7.28)	0.500	0.59(0.16;2.15)	0.423
Basic Laboratory data^c^								
Hemoglobin < 8	1.09(1.03;1.16)	**0.004** ^a^	3.21(1.46;7.07)	**0.004** ^a^	1.38(0.32;5.98)	0.663	1.21(0.54;2.74)	0.645
Hemoglobin [8–11]	1.01(0.95;1.07)	0.696	0.68(0.30;1.57)	0.366	0.56(0.13;2.4)	0.444	1.25(0.60;2.65)	0.552
Leucocytes < 4	1.07(0.95;1.22)	0.279	2.07(0.61;7.01)	0.241	0.59(0.018;19.90)	0.771	1.10(0.28;4.33)	0.90
Leucocytes≥12	0.98 (0.94;1.02)	0.361	1.15(0.69;1.91)	0.590	2.04(0.64;6.52)	0.227	0.65(0.38;1.10)	0.106

^a^ Statistically significant

^b^ **According to nurse’s appreciations**

^c^ hemoglobin value was g/dl; leucocyte value was 10^3^/μl

*BSI* Bloodstream infection, *GII* Gastro-intestinal infection, *UTI* Urinary tract infection

**Note** The different data were adjusted for gender, age and weigh during the multivariate analysis

*MUAC* Mid-Upper Arm Circumference

The multivariate logistical regression analysis revealed that a general bacterial infection was significant associated to the following clinical signs and symptoms: high axillary temperature ≥ 39.5 °C (*p* = 0.002), diarrhea (*p* = 0.003), edema (*p* = 0.010) and convulsion (*p* = 0.021) (See Table 3). Based on infection type, bacterial BSI was significantly associated with high axillary temperature ≥ 39.5 °C [p = 0.002; IC 95% = (1.51;5.97)], diarrhea [*p* = 0.019; IC 95% = (1.12;3.46)] and edema [*p* = 0.017; IC 95% = (1.38;26.39)]. Bacterial GII was not associated with clinical signs and symptoms, and basic laboratory data according to this second criteria. The multivariate analysis was adjusted for age, gender and weight, which are confounding factors.

**Table 3:** Clinical signs and symptoms and basic laboratory results associated to bacterial infection in multivariate logistic regression analysis

	Bacterial infected group		BSI		UTI		GII	
Clinical signs and symptoms	OR (IC 95%)	*P* value	OR (IC 95%)	*P* value	OR (IC 95%)	*P* value	OR (IC 95%)	*P* value
Z-score Weight/age < 2SD in kg/month, kg/age, mean (SD)	0.95(0.68;1.33)	0.761	0.82(0.67;1.01)	0.063	0.82(0.54;1.25)	0.358	0.87(0.72;1.05)	0.140
Z-score Height/age < 2SD in cm/month, cm/kg, mean (SD)	1.03(0.97;1.08)	0.372	1.02(0.97;1.07)	0.486	0.98(0.89;1.09)	0.768	1.02(0.97;1.07)	0.442
Diarrhea	1.84(1.22;2.78)	0.003*	1.97(1.12;3.46)	0.019*	3.97(1.12;14.02)	0.032*	1.36(0.8;2.3)	0.259
Dehydration	0.74(0.28;1.94)	0.538	0.65(0.22;1.98)	0.452	1.35(0.17;10.4)	0.775	0.58(0.09;3.51)	0.550
Edema	6.27(1.56;25.23)	0.010*	6.04(1.38;26.39)	0.017*	0.82(0.01;52.99)	0.926	2.2(0.28;17.39)	0.455
Convulsion	3.46(1.21;9.87)	0.021*	2.06(0.6;7.03)	0.248	0.68(0.02;29.4)	0.844	2.34(0.5;11.01)	0.281
Axillary temperature [39.5,42]	2.25(1.34;3.79)	0.002*	3.01(1.51;5.97)	0.002*	0.97(0.15;6.39)	0.975	1.41(0.68;2.9)	0.356
Pallor of conjunctiva	1.53(0.74;3.14)	0.249	1.31(0.55;3.15)	0.540	2.76(0.49;15.53)	0.250	1.01(0.31;3.31)	0.982
Splenomegaly	1.78(0.73;4.300)	0.638	2.28(0.9;5.77)	0.081	0.34(0.01;7.9)	0.501	1.17(0.25;5.53)	0.840
Hepatomegaly	1.17(0.50;2.75)	0.725	1.03(0.38;2.78)	0.951	0.25(0.01;5.15)	0.372	0.79(0.18;3.37)	0.746
Laboratory basic data^*^								
Hemoglobin < 8	1.34(0.68;2.62)	0.394	1.96(0.82;4.69)	0.133	0.95(0.2;4.63)	0.954	1.05(0.44;2.48)	0.913

*Statistically significant

^a^hemoglobin value was g/dl; leucocyte value was 10^3^/μl

*BSI* Bloodstream infection, *GII* Gastro-intestinal infection, *UTI* Urinary tract infection

**Note:** The different data were adjusted for gender, age and weigh for the multivariate analysis

The performance of clinical signs and symptoms significantly associated to bBSI were reported in Table 4. Clinical signs and symptoms associated to bacterial BSI after multivariate analysis were combined to determine their performance to predict BSI in febrile children under – 5 years. If we apply the combination “presence of diarrhea and edema” to determine whether an actual bacterial BSI is present, only one case of bacterial BSI out of 65 positive bacterial BSI (1.54%) detected by blood culture could be diagnosed. Three cases out of 65 positive bacterial BSI (4.62%) could be diagnosed if the combination “absence of diarrhea and presence of edema” was used.

**Table 4:** Performance of significant clinical variables in multivariate analysis to predict BSI in febrile children under-5 years of age

		Blood culture positive n (%)	Blood culture negative n (%)
Diarrhea+			
Diarrhea +	Yes	29(2.64)	342(31.12)
	No	36(3.27)	692(62.97)
Edema+			
Edema+	Yes	4(0.36)	6(0.55)
	No	61(5.55)	1028(93.54)
Diarrhea + edema +			
Diarrhea + edema +	Yes	1(0.09)	0(0)
	No	64(5.82)	1034(94.09)
Diarrhea - edema +			
Diarrhea - edema +	Yes	3(0.27)	6(0.55)
	No	62(5.64)	1028(93.54)
Diarrhea + edema -			
Diarrhea + edema -	Yes	28(2.55)	342(31.12)
	No	37(3.37)	692(62.97)
Diarrhea - edema -			
Diarrhea - edema -	Yes	33(3.00)	686(62.42)
	No	32(2.91)	348(31.67)

## Discussion

The present study showed that high fever (axillary temperature > 39.5 °C), diarrhea and edema were only associated with a bacterial BSI in febrile children under - 5 years of age. However, there is a risk to overlook a real bacterial BSI if only the clinical signs and symptoms combined to basic hematology data are used in the assessment of the child (only 1/65 case of BSI could be diagnosed if presence of edema and diarrhea should be considered). Although clinical signs and symptoms combined with basic hematological data are useful for suspicion of bacterial infections, there is a need to determine the presence of bacteria in clinical specimens related to the site of infection. Furthermore, there is a necessity to develop practical tools to distinguish bacterial infections from other infections in febrile children in order to be able to treat fatal, but treatable, diseases, in particular when a reliable diagnostic test would be available in an early stage of the infection or at the first contact with health professionals [21]. Therefore, we conclude on the basis of our data that clinical signs and symptoms, and basic hematology data cannot diagnose bacterial infections in febrile children under 5 years of age. As a consequence, this may lead to over prescription of antibiotics as attending health workers do not want to take the risk of missing a diagnosis. A definitive alternative to diagnose bacterial infections remains the development of practical laboratory tools, similar to malaria rapid diagnostic test, in other words cheap, fast and easy to perform without much training.

Previous study demonstrated that malaria may predispose to non-typhoid salmonella (NTS) bacteremia [22, 23]. In the present study, malaria prevalence was 50% and *Salmonella enterica* (80.5% of positive blood culture and 50% of stool culture) was the main species isolated from blood and stool culture [18, 24–26]. Therefore, NTS should always be considered in the case of a suspected bacterial infection in a malaria endemic area.

Studies by Mtove et al. [27] and Brent et al. [28] have both demonstrated a relative low positivity of blood stream infection in African children who do not meet indications for hospital admission. Conversely, other studies on blood stream bacterial infections in African children admitted to hospital have demonstrated that bloodstream or cerebral-spinal fluid (csf) bacterial infections are relatively common in children admitted to hospital and significantly influence mortality [29, 30]. In our study, it was found that children who meet indications for hospital admission were more prevalent in the group of patients with bacterial infections than those without a bacterial infection. However, almost 70% of children with bacterial infections in general and 55% of children with positive bacterial BSI did not meet indications for hospital admission. Health workers should therefore pay more attention to children who did not meet indications for hospital admission at the first contact, in particular when a malaria infection can be excluded.

Our study confirms the limitation of using clinical signs and symptoms, and basic laboratory data to diagnose bacterial infections [31, 32]. In general, only severe cases of bacterial infections are reported to be diagnosed by clinical signs and symptoms in young children [33, 34]. In our study the combination of presence of diarrhea and edema (associate to BSI after multivariate analysis) to diagnose bBSI leaded to miss important part of bBSI. Nonetheless, high axillary temperature remains an indicator of bBSI [35, 36]. Therefore, it is unlikely to save the lives of febrile children based on clinical signs and symptoms, and basic hematology data if bacterial diagnosis cannot be done at the early stage of infections. The availability of practical diagnostic tools for screening could be a good solution to save time and reduce the risk of fatal issues in the management of fever in this age group [21].

Although the multivariate analysis showed an association between UTI and diarrhea, the low prevalence of UTI deserve more attention. Previous studies reported association between UTI with high axillary temperature, sex and age (12–35 months) [37]. However, urine sample collection deserves minimum hygiene condition. In the present study, stool and urine were systematically collected for each participant by parent/guardian and *E. coli* was the only specie isolated. Maybe the urine samples were contaminated with stool during urine sample collection.

Bacterial GII was not associated to any clinical signs and symptoms, and also basic hematology data. This is in line with the general observation that bacterial GII is not associated with fever [38–42].

A limitation of our study is that it does not present information on bacterial respiratory tract infections. The Pneumonia Etiology Research for Child Health (PERCH) project, a multi-country, case-control study to determine the etiology of and risk factors for (very) severe pneumonia in young children (1–59 months of age), provides a wealth of information on respiratory infection in African children [43]. These studies have demonstrated that nasopharyngeal carriage of *S. pneumonia* and *S. aureus* is very common in healthy children [44] but their role as potential fever causing pathogen was not further studied here as such research would require a case-control design, which is beyond the scope of the present work.

## Conclusions

Despite the usefulness of clinical signs and symptoms in combination with basic hematology data in detecting bacterial blood stream infections, our study demonstrated the necessity to confirm the presence of bacterial infection with practical diagnostic tools. Nevertheless, the worldwide concern about the over prescriptions of antibiotics cannot be circumvented if clinical signs and symptoms, combining to basic hematology data remain the only information available in area without laboratory facilities. The development of practical and easy deployable diagnostic tools to diagnose bacterial infections remains a priority.

## Abbreviations

API: Analytical Profile Index
BD: Becton Dickinson
BSI: Bloodstream Infection
CFU: Colonies forming units
CG: Chocolate Gelose
CLED: Cysteine Lactose Electrolyte Deficient
CMA: Centre Médical avec Antenne Chirurgicale
CO_2_: Carbon dioxide
CRF: Case Report Form
CRUN: Clinical Research Unit of Nanoro
CSF: Cerebral-spinal fluid
EDTA: Ethylene-Diamine Tetra-Acetic acid
EMB: Eosin-Methylene Blue
EPI: Extended Program Immunization
GII: Gastro-Intestinal Infection
IVX: IsoVitalex
MUAC: Mid - Upper Arm Circumference
NICD: National Institute for Communicable Diseases
NPV: Negative Predictive Value
NTS: Non-Typhoid Salmonella
PERCH: Pneumonia Etiology Research for Child Health
PPV: Positive Predictive Value
RDT: Rapid Diagnostic Test
SB: Sheep Blood
SS: Salmonella and Shigella
UTI: Urinary Tract Infection
WHO: World Health Organization
